# The combined effects of motor and social goals on the kinematics of object-directed motor action

**DOI:** 10.1038/s41598-020-63314-y

**Published:** 2020-04-14

**Authors:** Maria Francesca Gigliotti, Adriana Sampaio, Angela Bartolo, Yann Coello

**Affiliations:** 1 Univ. Lille, CNRS, UMR 9193 - SCALab - Sciences Cognitives et Sciences Affectives, F-59000 Lille, France; 2 Psychological Neuroscience Lab, Research Center in Psychology (CIPsi), School of Psychology, University of Minho, Campus de Gualtar, 4710-057 Braga, Portugal

**Keywords:** Social behaviour, Human behaviour

## Abstract

Voluntary actions towards manipulable objects are usually performed with a particular motor goal (i.e., a task-specific object-target-effector interaction) and in a particular social context (i.e., who would benefit from these actions), but the mutual influence of these two constraints has not yet been properly studied. For this purpose, we asked participants to grasp an object and place it on either a small or large target in relation to Fitts’ law (motor goal). This first action prepared them for a second grasp-to-place action which was performed under temporal constraints, either by the participants themselves or by a confederate (social goal). Kinematic analysis of the first preparatory grasp-to-place action showed that, while deceleration time was impacted by the motor goal, peak velocity was influenced by the social goal. Movement duration and trajectory height were modulated by both goals, the effect of the social goal being attenuated by the effect of the motor goal. Overall, these results suggest that both motor and social constraints influence the characteristics of object-oriented actions, with effects that combine in a hierarchical way.

## Introduction

The planning and monitoring of an object-directed motor action (also referred to as transitive action) depend on the processing of various factors, related to both the object and the agent of the motor action. These include the intrinsic characteristics of the object such as its shape, size, weight or texture^1–5^, its extrinsic features, such as its spatial location, orientation and distance from the agent’s body^6–8^, and the final posture of the limb used for the motor action (i.e., the end-state comfort effect^9^). These factors influence various features of the ongoing motor action, including the kinematics of the approach movement and the grasping of the object. For instance, object size and distance modulate arm velocity and aperture of the grip, and they also shape the posture of the hand and fingers on the object, thus allowing for a correct grasp^10–14^.

In addition to the physical characteristics of manipulable objects, the motor goal of the action (i.e., the task-specific object-target-effector interaction) also influences the kinematic features of an object-directed motor action. This has been well documented in tasks modifying the physical characteristics of the motor target (intended as the final spatial location), such as its distance or size (Fitt’s law^15^). In this respect, the main finding is that movement duration concurrently increases with the reduction in target size or the increase in target distance (in relation to the resulting index of difficulty^15^). Furthermore, the pioneering work by Marteniuk, Mackenzie, Jeannerod, Athenes and Dougas in 1987^16^ revealed that what people intend to do with the object after having grasped it (e.g., grasp-to-throw or grasp-to-place) influences the kinematic pattern of the grasping action. The effect of the motor goal on the spatio-temporal features of motor performance was later confirmed in different grasping tasks^10,17–19^, and extended to pointing^20^, writing^21^ and even communicative gesturing^22,23^. It was further shown that observing an object-directed motor action provides the means to anticipate the underlying motor goal through spatio-temporal variations in task execution, well before the action is fully completed, so that its effects can be anticipated^24,25^.

More recently, the social goal of an object-directed motor action was also found to influence movement kinematics (for reviews see^26–30^). A number of studies have indeed revealed that an object-oriented motor action performed with a social goal, i.e. intending to influence the behavior of another person^31^, is characterized by a slower velocity and a higher arm trajectory^32–36^. It was suggested that such spatio-temporal deviants render the movement more salient and more likely to capture the eye-gaze and attention of the confederate involved in the interaction^37,38^. For instance, exploring a cooperative motor task, Quesque and Coello^38^ reported that the spatial amplification of a grasp-to-place motor action was broader, resulting in a higher arm trajectory, when the partner’s eye-level was set at a higher position. This result is in line with the key role of the gaze in the process of action understanding in social contexts^39–43^. Because of their social value, the spatio-temporal variations of object-directed motor actions are also thought to serve as crucial cues for an observer to identify the agent’s social goal^44–46^. The perception of such spatio-temporal deviants induced by the social context would allow an observer to prepare appropriate motor responses, thus contributing to the achievement of a shared objective^35,47^ and the improvement of social interactions^34,48^. However, it was found that the detection of a social goal from motor deviants also depends on the observer’s cognitive social abilities^49^ and is facilitated by the presence of contextual environmental cues^50^.

Despite the wealth of studies that have highlighted the key role of motor and social goals in motor performances, no study has yet examined the effect of concurrently manipulating these two independent goals in an object-directed motor task. Moreover, the way in which the social goal was manipulated in previous studies did not help easily dissociate its contribution to motor actions from that of the motor goal. This was the case when the social goal consisted in grasping an object and placing it in the hand of a confederate instead of a physical container^10,32,51,52^. Although using the partner’s hand as a target for the motor action altered the kinematics of the placing phase of a grasp-to-place action, suggesting an influence of social intention, the effects observed in such a situation could be the result of either the social nature of the task (social goal) or the modification of the physical characteristics of the target (motor goal).

Therefore, the combined effects of motor and social goals on the kinematics of a voluntary motor action remain an open issue. In the present study, we tackled this issue by developing a paradigm for assessing the specific effects of motor and social goals on the execution of an object-directed motor action. We designed a task involving a dyad of participants, which consisted in performing two successive grasp-to-place actions. The first action (named “preparatory action”) was always performed by the same participant and consisted in grasping a wooden object in order to place it on either a small or large circle used as a spatial target (motor goal), and located in the middle of the workspace. This first action prepared participants for the second action, which could be performed by either the same participant or the confederate (social goal). This second action (named “main action”) consisted in grasping and placing the same wooden object on a sideway spatial target (either a small or large circle) under temporal constraint and with feedback about motor performances. Hence, we manipulated (1) the motor goal of the task by modifying the size of the target in accordance with Fitts’ law^15^ (index of difficulty of 2 vs 3 bits), and (2) the social goal of the task by changing the agent performing the second grasp-to-place action (main action), in accordance with the paradigm developed by Quesque *et al*.^35^. By combining the motor goal and the social goal in such a way, we were able to probe their respective contribution as well as their interaction in an object-directed motor task. For the purpose of the present study, we focused our analysis on the variation in the temporal and kinematic parameters of the preparatory action only. Indeed, the main action served mainly to create a cooperative context for the task, as well as to orient the participants’ attention on this part of the task, so that they behaved spontaneously in the preparatory action, which was at the core of the study. More specifically, we analyzed movement duration, peak wrist velocity, the percentage of time taken by the deceleration phase and the peak wrist elevation (as an index of the height of the trajectory). These analyses were performed for both the object grasping and placing phases constituting the preparatory action. In line with the above-mentioned literature, our main expectations for both the grasping and placing phases were that 1) movement time as well as trajectory height should increase and movement velocity should decrease when the preparatory action fulfils a social goal; 2) movement time and deceleration phase should increase when the motor goal of the preparatory action decreases in size; 3) the effect of the social goal on motor kinematics should interact with the effect of the motor goal, but only in the placing phase.

## Results

**Figure 1:**
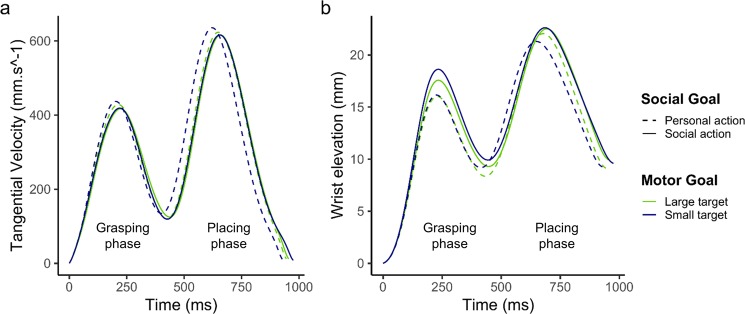
(**a**) Mean velocity and **(b**) trajectory height profiles as a function of Social (Personal action, Social action) and Motor (Small target, Large target) goal. The two bell-shaped curves represent the grasping and placing phases of the preparatory action, each characterized by (**a**) a velocity peak and (**b**) a height peak.

The temporal and kinematic parameters were computed and analyzed separately for the grasping and placing phases of the preparatory action. Mean values and standard deviations for each parameter are reported in Table 1 as a function of the experimental condition and action phase. In addition, Fig. 1 shows mean velocity and trajectory height profiles of the preparatory action (including both the placing and the grasping phases) as a function of the experimental conditions.

**Table 1:** Mean values (and standard deviations) of each kinematic parameter as a function of the phase, Motor goal and Social goal.

Condition	*N*	Kinematic Parameter			
		Movement time (ms)	Percentage of deceleration time (%)	Peak wrist velocity (mm.s ^− 1^)	Peak wrist elevation (mm)
***Grasping phase***					
Personal-Small	525	408.65 (79.67)	49.33 (8.65)	494.08 (97.37)	46.64 (10.34)
Social-Small	496	435.29 (84.09)	49.41 (8.29)	475.50 (102.06)	49.56 (10.74)
Personal-Large	519	427.24 (81.61)	49.50 (8.10)	488.43 (104.31)	47.05 (10.08)
Social-Large	484	439.32 (83.44)	49.00 (8.63)	473.17 (105.32)	48.20 (9.17)
***Placing phase***					
Personal-Small	525	523.44 (91.10)	59.37 (5.55)	702.29 (96.06)	52.07 (12.07)
Social-Small	496	542.43 (86.72)	59.60 (5.44)	675.25 (81.22)	53.31 (12.26)
Personal-Large	519	517.10 (92.81)	57.60 (5.91)	690.09 (91.84)	52.75 (11.99)
Social-Large	484	516.33 (87.79)	57.81 (5.55)	664.92 (79.99)	52.90 (11.58)

“N” indicates the number of movements in each condition.

### Temporal and kinematic analysis

#### Movement time

Concerning the grasping phase, the conditional coefficient of determination of the model (normal distribution, see Table 2) was 0.62. Statistical analysis showed no effect of Motor goal (estimate = −10.430, *SE* = 6.14, *χ2(1*) = 2.88, *p* = 0.089), but an effect of the Social goal (estimate = −22.329, *SE* = 5.67, *χ2(1)* = 15.48, *p* < 0.001), with a longer movement time characterizing the social compared to the personal action. The Motor goal x Social goal interaction was also significant (estimate = −16.315, *SE* = 4.61, *χ2(1)* = 12.51, *p* < 0.001), with the difference in movement time between the social and personal action being greater with the small target than with the large one. Multiple comparison analysis revealed that movement time was longer for social compared to personal action when acting towards both the large (estimate = 14.20, *SE* = 6.14, *t.ratio* = 2.31, *p* = 0.013) and the small target (estimate = 30.50, *SE* = 6.11, *t.ratio* = 4.98, *p* < 0.001).

**Table 2:** Family distribution, link function, fixed effects and random effects specified in the model as a function of the kinematic parameter analyzed.

Kinematic parameter	Family distribution	Link function	Fixed effects	Random effects
***Grasping phase***			Social goal + Motor goal + Social goal * Motor goal	Social goal + Motor goal | Participant
Movement Time	Gaussian	Identity*		
Percentage of deceleration time	Gaussian	Identity*		
Peak wrist velocity	Gaussian	Identity*		
Peak wrist elevation	Gaussian	Log		
***Placing phase***				
Movement Time	Gaussian	Log		
Percentage of deceleration time	Gaussian	Identity*		
Peak wrist velocity	Gaussian	Identity*		
Peak wrist elevation	Gaussian	Log		

The family distribution refers to the distribution of the dependent variable. The link function consists in the mathematical function characterizing the relationship between the fixed factors and the dependent variable. The elements before and after (|) refer to the random slopes and random intercepts respectively. ^*^Glmer function used with a Gaussian distribution and a link “identity” corresponds to a linear mixed-effect model.

Regarding the placing phase, the conditional coefficient of determination of the model (log-normal distribution, see Table 2) was 0.02. The effect of Motor goal was significant (estimate = 0.035, *SE* = 0.01, *χ2(1)* = 7.52, *p* = 0.006), with longer movement time for the small than the large target, but the effect of Social goal was not (estimate = −0.026, *SE* = 0.01, *χ2(1)* = 3.60, *p* = 0.058). The Motor goal x Social goal interaction was significant (estimate = −0.041, *SE* = 0.01, *χ2(1)* = 15.66*, p* < 0.001), due to longer movement time for the social compared to the personal action in the presence of the small (estimate = 0.044, *SE* = 0.01, *z.ratio* = 3.12, *p* < 0.001) but not the large target (estimate = 0.004, *SE* = 0.01, *z.ratio* = 0.27, *p* = 0.395).

#### Percentage of deceleration time

Concerning the grasping phase, the conditional coefficient of determination of the model (normal distribution, see Table 2) was 0.34. The effect of Motor goal was not significant (estimate = 0.0002, *SE* = 0.005, *χ2(1)* = 0.001, *p* = 0.972), nor was the effect of the Social goal (estimate = 0.0005, *SE* = 0.005, *χ2(1)* = 0.011, *p* = 0.916). The Motor goal x Social goal interaction was not significant either (estimate = −0.005, *SE* = 0.006, *χ2(1) =* 0.780, *p* = 0.377).

As regards the placing phase, the conditional coefficient of determination of the model (normal distribution, see Table 2) was 0.30. In contrast with the grasping phase, the effect of Motor goal was significant (estimate = 0.017, *SE* = 0.004, *χ2(1)* = 15.34, *p* < 0.001), with a longer deceleration phase for actions towards the small target than towards the large one. The effect of Social goal was not significant (estimate = −0.004, *SE* = 0.003, *χ2(1)* = 1.15, *p* = 0.283), nor was the Motor goal x Social goal interaction (estimate = 0.001, *SE* = 0.004*, χ2(1)* = 0.08, *p* = 0.771).

#### Peak wrist velocity

Concerning the grasping phase, the conditional coefficient of determination of the model (normal distribution, see Table 2) was 0.67. The effect of Motor goal was not significant (estimate = 2.930, *SE* = 7.17, *χ2(1)* = 0.17, *p* = 0.683), while the effect of Social goal was (estimate = 17.437, *SE* = 6.93, *χ2(1)* = 6.33, *p* = 0.012), with the personal action being performed with a higher velocity than the social action. This effect was not modulated by the Motor goal, as the Motor goal x Social goal interaction was not significant (estimate = 4.092, *SE* = 5.30, *χ2(1)* = 0.59, *p* = 0.440).

As regards the placing phase, the conditional coefficient of determination of the model (normal distribution, see Table 2) was 0.61. As for the grasping phase, the effect of Motor goal was not significant (estimate = 10.465, *SE* = 7.55, *χ2(1)* = 1.92, *p* = 0.166), while the effect of Social goal was significant (estimate = 28.377, *SE* = 5.92, *χ2(1)* = 22.99, *p* < 0.001), with the personal action reaching a higher velocity than the social action. Again, the Motor goal x Social goal interaction was not significant (estimate = 4.093, *SE* = 5.05, *χ2(1)* = 0.66*, p* = 0.417).

#### Peak wrist elevation

Regarding the height of the trajectory during the grasping phase, the conditional coefficient of determination of the model (log-normal distribution, see Table 2) was 0.03. The effect of Motor goal was not significant (estimate = 0.010, *SE* = 0.01, *χ2(1)* = 0.67, *p* = 0.413), while the effect of Social goal was (estimate = −0.040, *SE* = 0.006*, χ2(1) =* 51.13*, p* < 0.001), the social action being characterized by a higher trajectory than the personal action. This effect was modulated by the Motor goal, as revealed by the significant Motor goal x Social goal interaction (estimate = −0.04, *SE* = *0*.01*, χ2(1) =* 17.76*, p* < 0.001). In fact, the difference in wrist elevation between the social and personal action was greater with the small target than with the large one. Multiple comparisons showed that the increased wrist elevation characterizing the social action compared to the personal action was significant with both the small target (estimate = 0.06, *SE* = 0.007, *z.ratio* = 8.22, *p* < 0.001) and the large one (estimate = 0.02, *SE* = 0.007, *z.ratio* = 2.603, *p* = 0.005).

As regards the placing phase, the conditional coefficient of determination of the model (log-normal distribution, see Table 2) was 0.03. The effect of Motor goal was not significant (estimate = −0.003, *SE* = 0.01*, χ2(1) =* 0.05*, p* = *0*.816), nor was the effect of Social goal (estimate = −0.01, *SE* = 0.01*, χ2(1) =* 0.62*, p* = 0.431), contrasting with the grasping phase. However, the Motor goal x Social goal interaction was significant (estimate = −0.025, *SE* = 0.009*, χ2(1) =* 6.89*, p* = 0.009), owing to a greater difference in peak wrist elevation between the social and personal action with the small target than with the large one. Multiple comparisons showed that the increase in wrist elevation in the social action compared to personal action was significant with the small target (estimate = 0.02, *SE* = 0.01, *z.ratio* = 1.66, *p* = 0.048) but not with the large one (estimate = −0.002, *SE* = 0.01, *z.ratio* = −0.18, *p* = 0.572).

## Discussion

Previous studies on object-directed motor action have shown that motor performances are influenced by either the motor goal of the action (i.e., the constraints associated with the object-target-effector system) or the social goal of the action (i.e., the person who would benefit from this particular object-directed motor action)^8,10,14,16,34,35^. Within this context, the aim and the novelty of the present study was to study the combined effects of motor and social goals when concurrently involved in an object-directed motor task. By analyzing the temporal and kinematic features of object grasping and placing phases, we observed that motor and social goals have dissociated effects on the spatio-temporal features of object-directed motor actions, which are summarized and discussed below.

The first important finding was that the analysis of trajectory height and movement duration revealed an interaction between the effects of motor and social goals. Confirming previous reports^32–35,38,47^, we found that object-directed actions performed with a social purpose were characterized by a slightly more curved path (on average 1 and 2 mm higher in the grasping and placing phases respectively) and a longer duration (on average 22 and 9 ms in the grasping and placing phases respectively) compared to object-directed actions performed with a personal purpose. However, the novel finding was that, in both the grasping and placing phases, the effect of the social goal on trajectory height and movement duration was modulated by the motor goal. More specifically, the difference in trajectory height caused by the social goal of the task was greater when the grasp-to-place action involved a small target, while it was smaller (grasping phase) or even absent (placing phase) when it involved a large target. This original result underlines that the effect of the social goal on an object-directed motor action, reported in previous studies^32,34,35,38,47^, depends on the constraints associated with the object-target-effector system, and therefore on the motor goal of the action. In our study, the reduced effect of the social goal observed in the presence of the large target could be related to the low-level constraints associated with Fitts’ law^15^. More specifically, the increase in target size induced a decrease in the index of difficulty (from 3 to 2 in our task for small and large targets respectively) resulting in the performance of faster movements (i.e. characterized by a shorter duration). As a consequence, the faster the movement, the less time participants have to adapt the height of their arm trajectory in relation to the social goal of the task. This interference effect was particularly visible in the placing phase in which, owing to the characteristics of the experimental paradigm used, both the motor and social goals affected the motor performance. In contrast, this interference was less notable in the grasping phase, where only the social goal could directly influence the motor performance. A similar pattern of results emerged from the analysis of movement duration. Data showed that motor and social goals interacted, with a larger increase in movement duration for the social action in the presence of the small target than the large one. Again, in the presence of the large target, the difference in movement duration between the social and personal action was smaller in the grasping phase and absent in the placing phase. Taken as a whole, the interaction effects that emerged from the analysis of temporal and spatial features of the grasp-to-place action showed a modulatory effect of the motor goal on the influence of the social goal. This suggests that the features of the physical target, and more likely the constraints associated with the object-target-effector system, prevail over the social constraints of the task when both a motor and a social goal concurrently determine the spatio-temporal characteristics of the object-directed motor action.

The second important finding of the present study was the observation of differential effects of motor and social goals on the kinematic parameters of movements. Surprisingly, although the variation in movement duration was induced by both the motor and social goals, we did not observe any effect of the motor goal on the maximum velocity reached during the grasp-to-place action. As a possible explanation, we speculate that Fitts’ law did not alter movement acceleration as much as the deceleration phase, for which we found an effect of target size. This observation fits well with previous studies which reported that target size mainly impacts the deceleration phase of a motor action, the latter lasting longer when the motor action is directed towards a small target^53–55^. By contrast, and in line with the existing literature^23,33,35,47^, we found an effect of the social goal on maximum velocity, with the personal action being performed faster compared to the social action (on average 17 and 28 mm.s^−1^ in the grasping and placing phases respectively). However, when analyzing the deceleration phase, we found no effect of the social goal on either the grasping or the placing phase. This absence of effect contradicts previous findings that showed that motor actions performed with a social goal are characterized by a longer deceleration phase compared to motor actions performed with a personal goal^23,33,37^. This discrepancy between the present and previous results may stem from the combined effect of motor and social goals (when concurrently involved). In contrast to other studies, the present paradigm was conceived to dissociate the effects of motor and social goals. We thus tested the effect of the social goal while keeping constant the constraints associated with the object-target-effector system (i.e., using a small or a large target for either the personal or the social action). Therefore, the effect of the social goal on the deceleration phase found in previous studies could have resulted from the fact that the final motor target used to trigger the grasping action was not kept uniform across the conditions, opposing for instance the hand of a confederate to a physical support^10,32,51,52^. To confirm this interpretation, future studies should replicate and extend the present findings using different paradigms based on ecological social tasks implying different kinds of movement synchronization.

## Conclusion

To our knowledge, the present study represents the first experimental work investigating the combined effects of motor and social goals on the execution of object-directed motor action. The results showed that social and motor goals have an impact on specific kinematic parameters of the object-directed motor action (i.e., deceleration phase is affected only by the motor goal, while peak velocity is affected only by the social goal), as well as combined effects on other kinematic parameters (i.e., trajectory height and movement duration). More importantly, these combined effects reflect a reduction in the effect of the social goal in relation to the motor goal. These original findings suggest the existence of a hierarchy between motor and social constraints, the first taking precedence over the second. However, more investigation using different paradigms and social tasks would be required in order to confirm and extend the present findings. Furthermore, the present study suggests that the effects of motor and social goals might be sensitive to the paradigm used, and cautions against potential biases that could emerge from a lack of consideration in experimental tasks. Finally, the present findings pave the way for further research on object-directed motor actions performed in social contexts for the study of interactions, both in natural conditions and in environments involving artificial agents.

## Method

### Participants

Twenty-eight healthy participants voluntarily took part in the experiment (twenty-four females, 18–35 years old, *M* = 23.36 years, *SD* = 6.60 years). They were right-handed, as assessed by the Edinburgh Handedness Inventory^56^ (mean laterality quotient = 0.86, *SD* = 0.17), and declared having normal or corrected-to-normal visual acuity and no perceptual or motor deficit. They had no prior information about the hypotheses tested in the study and gave their written informed consent prior to the beginning of the experiment. The protocol was approved by the ethical committee in behavioral sciences of the University of Lille (Ref. Number 2019–363-S75) and was conducted in conformity with the ethical principles of the Declaration of Helsinki^57^.

### Confederates

Three female confederates of the experimenter (right-handed, aged 24, 25 and 27 years old) took part in the experiment, performing the task in cooperation with the participants and behaving as naïve participants.

### Stimuli

**Figure 2:**
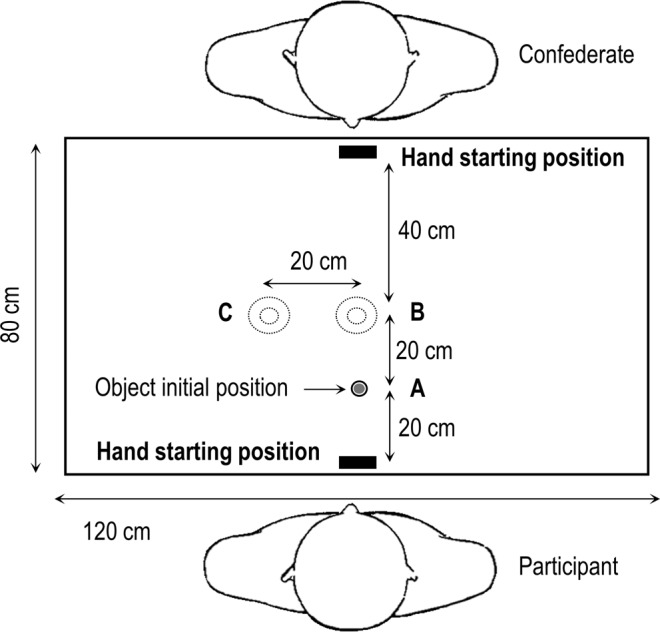
Representation of experimental setup. The two dotted circular landmarks B and C represent the small and large targets used in the task. At the beginning of each trial, the object (filled grey circle) was placed on landmark A.

The experimental setup (see Fig. 2) involved a 120 × 80 cm table covered by a 120 × 80 cm black, non-reflecting cloth. The task consisted in grasping and placing a wooden object (diameter 1.7 cm, thickness 1 cm) from landmark A to landmark B (preparatory action), then from landmark B to landmark C (main action). Landmarks were represented on the covering cloth by black circles. Landmark A served as the initial position for the object, while landmarks B and C served as targets for the task. The diameter of landmark A was 1.7 cm. The diameter of landmarks B and C were either 5 cm (small targets) or 10 cm (large targets). These two target sizes were chosen according to Fitts’ law^15^, using the formula:

1 $$ID=lo{g}_{2}\left(\frac{2D}{W}\right)$$

where *ID* stands for “index of difficulty” (in bits), *D* indicates the distance to the center of the target (20 cm in the present study) and *W* the width (size) of the target (5 or 10 cm of diameter in the present study). The index of difficulty was 3 bits for the small target and 2 bits for the large target. All inter-target distances were 20 cm. Small and large targets were presented using two different covering cloths, which were alternately fixed on the table. In addition to landmarks A, B and C, two white rectangular landmarks located on the opposite edges of the table were used to indicate the participant’s hand starting position.

### Procedure

**Figure 3:**
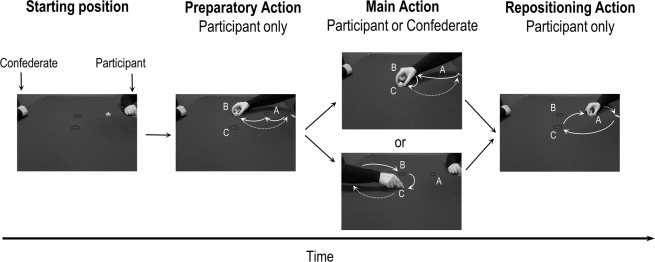
Sequential order of three grasp-to-place motor actions composing the task. At the end of each action (preparatory, main and repositioning), participants repositioned their hand at the starting position (dotted arrows).

The task was derived from the paradigm developed by Quesque *et al*. (2013)^35^. During the experiment, the participant sat on one side of the table in front of a confederate. The latter was randomly chosen between one of the three accomplices of the experimenter but pretended to be a naive participant. In order to avoid an effect of confederate’s eye-level^38^, the chair where the confederate sat was adjusted so that the eye-levels of confederates and participants were similar. In each trial, the participant was required to move the wooden object from one target to another following a specific sequence of three grasp-to-place actions: the *preparatory action*, the *main action* and the *repositioning action* (see Fig. 3). The *preparatory action* was always performed by the participant and consisted in moving the wooden object from its initial position A to target B. The *main action* was presented to participants as the core action of the task; it could be performed by the participant or the confederate and involved moving the wooden object from target B to target C. We used the term *main action* as we wanted the participants to maintain their attention on this part of the task and not the preparatory action, so that the latter would be performed in a spontaneous way. Finally, the *repositioning action* was always performed by the participant, and consisted in moving the wooden object from target C to the initial position A, in order to get ready for the following trial. At the end of each action in the sequence, the participant and the confederate placed their hands in the starting position, with their thumb and index finger pinched together.

During the preparatory and the main actions, the participant was asked to place the wooden object within the circumference of the targets as precisely as possible. The error margin was established by subtracting the diameter of the wooden object from the radius of the target. As a consequence, the wooden object was considered as correctly placed when the difference between the center of the wooden object and the center of the target was not greater than 0.8 cm for the small targets and 3 cm for the large targets. During the main action only, the participant was requested to move the wooden object as fast as possible, moving the wrist at a velocity greater than 1040 mm.s^−1^ when grasping the wooden object. This velocity threshold corresponded to 80% of 1300 mm.s^−1^, which was the median velocity for both the small and large targets registered in a pilot study including 10 participants.

Each grasp-to-place action was triggered by an auditory cue and the participant had 2 s to perform the required action (preparatory, main or repositioning). When the accuracy and velocity constraints were met, the participant obtained one point and a sound of clinking coins was provided. Otherwise, an error sound was emitted, indicating that no points had been obtained. The sound of clinking coins and the error sound also triggered the repositioning action. The delays separating each action and each trial were randomized in order to prevent participants from adopting anticipatory strategies. The delay separating the end of the preparatory action and the auditory cue for the main action varied randomly between 1.5 and 2 s. The delay between the end of the main action and the auditory cue for the repositioning action was set at 2 s. The inter-trial delay varied randomly between 3 and 3.5 s.

Participants performed the task in four conditions, resulting from the combination of two experimental factors: Social goal (Personal action, Social action) and Motor goal (Small target, Large target). The Motor goal factor referred to the size (Small target, Large target) of B and C targets. The Social goal factor referred to whom the preparatory action was executed for, that is, moving the wooden object from target A to target B for a subsequent personal (Personal action) or confederate’s use (Social action) in the main action. The four resulting conditions were thus: Personal-Small, Social-Small, Personal-Large and Social-Large. Each condition was performed in a separated experimental block. The order of presentation of the four blocks was pseudo-randomized: the experiment could start by either the small or the large target and by either the personal or the social action. Participants switched to the other target only once they had performed both the social and the personal actions for one target.

The experimental session started with a training phase of 10 trials (10 sequences of the three grasp-to-place actions). The main action was performed by the participant during the first five trials and by the confederate during the last five trials. The experimental phase then involved the above-mentioned four blocks of trials. Each block ended when participants had won 20 points, i.e. when having performed 20 correct trials satisfying both the temporal and precision constraints of the main action. To check the validity of the experimental design, we calculated the number of incorrect trials depending on the motor goal (Small target, Large target), irrespective of the social goal (Personal action, Social action). As expected and in line with Fitts’ law, participants’ performances were characterized by more errors with the small (378 errors) than with the large target (183 errors).

### Data recording

Participants’ motor performances were recorded using the Qualisys motion analysis system, through three Oqus infrared cameras (sampling rate 200 Hz). During each movement, the three cameras tracked the 3D coordinates in space (*x*, *y*, *z*) of five passive markers placed on the participant’s right hand, more specifically on the index tip, the index base, the thumb tip and the scaphoid and pisiform bones of the wrist. No markers were placed on the hand of the confederate. A sixth marker was placed on the top of the wooden object in order to analyze its position in relation to the targets and check for precision. The cameras were calibrated at the beginning of each experimental session. A calibration was considered satisfactory when the system reached a standard deviation accuracy below 0.2 mm. Finally, each time the covering cloth was changed (to switch from small to large targets and vice versa), the *x*, *y*, *z* coordinates of the center of targets B and C were detected and calibrated, in order to obtain stable spatial references when evaluating the compliance with precision constraints.

### Data processing and statistical analysis

Motor performances recorded by the Qualisys system were processed by an in-house script adapted from the RTMocap toolbox for Matlab^58^. In line with the existing literature^34,35,38^, we analyzed only the trajectory of the wrist marker placed at the level of the scaphoid, which expresses arm movements without including wrist rotation. Each action was composed of two phases: the grasping phase and the placing phase. Action onset was considered as the first moment when the wrist marker reached 20 mm.s^−1^ ^35^. Action end corresponded to the moment when the wrist marker reached 20 mm.s^−1^ following peak velocity^35^. In the event that this threshold was not reached, the local minima following peak velocity was considered as the action end. For the purpose of the current study, only the parameters recorded during the preparatory action were considered. For both the grasping and the placing phases of the preparatory action, the analyses were carried out on the following kinematic parameters:

1. *Movement time (ms):* time elapsed between movement onset and movement end.
2. *Percentage of deceleration time (%)*: difference between movement time and time elapsed between movement onset and peak velocity, divided by movement time and multiplied by 100.
3. *Peak wrist velocity (mm.s*^−1^): maximum velocity reached by wrist in grasping and placing phases.
4. *Peak wrist elevation (mm)*: trajectory height corresponding to the maximum z (vertical) coordinate of wrist in grasping and placing phases.

These temporal and kinematic parameters were computed but excluded from further analysis if the movement was not correctly executed (i.e., impossibility to detect at least two local minima and/or two local maxima in the trajectory analysis), or if the reaction time (RT) was below 180 ms or above 2.5 standard deviations from the mean. Among the initial 2140 movements, 116 were removed from the data set, resulting in a loss of 5.42% of the data.

Statistical analyses and plots were performed with R version 3.5.1 (R Core Team 2018) and R Studio version 1.1.456. Each parameter of interest was analyzed as a function of the Motor goal (Small target, Large target) and Social goal (Personal action, Social action) using a mixed effects model approach. Mixed effects models are used to study the effect of experimental factors (called *fixed-effects parameters*) on the variable of interest, while taking into account the possible influence of other sources (referred as *random effects parameters*, e.g., inter-individual differences in sensitivity to the variables). Mixed effects models are particularly relevant for repeated measures experimental plans, as they can handle missing data and provide parameter estimates with acceptable type-I and type-II errors^59,60^. In the present study, we specifically fitted each parameter dataset with a generalized linear mixed effects model, using the glmer function of the lme4 1.1–21 package^61^.

For each of the kinematic parameters mentioned above, we applied a model incorporating Motor goal (Small target, Large target) and Social goal (Personal action, Social action) as fixed effects, including both main effects and the interaction effect. As random effects, our model included a by-subject random intercept and by-subject random slopes for the effect of Motor and Social goals. A by-confederate random intercept was not added in the final random structure of the model, as its specification did not statistically improve the model. Therefore, such a random effects structure was chosen on the basis of a compromise between the most complete random effect structure^60^ and the optimal random effect structure supported by the data^62,63^, in order to avoid model over-parametrization.

In addition to the fixed and random effects, glmer requires users to specify the type of error distribution. For this purpose, we analyzed the distribution followed by the response variable and residuals (separately for each kinematic parameter), by computing kurtosis and skewness and visually analyzing the distribution through histograms and Q-Q plots. Specifically, we fitted a Gaussian distribution to the peak wrist velocity and the percentage of deceleration time datasets for both grasping and placing phases, and to the movement time dataset for the grasping phase only. We fitted a log-normal distribution to the peak wrist elevation datasets for both grasping and placing phases, and to the movement time dataset for the placing phase only (see Table 2).

The model parameters (relating to fixed effects and random effects) were estimated by Laplace approximation and statistically tested with Wald’s *χ2*. The conditional coefficient of determination *R*^2^ (R_GLMM_(c)_²) quantifying the proportion of variance explained by the model (including both random and fixed effects, see^64^) was calculated using the function r.squaredGLMM of the MuMIn package version 1.46.6^65^. We reported the conditional coefficient of determination R^2^ obtained from generalized mixed models although their use and interpretation are considered controversial^66^. Finally, we performed one-tailed pairwise multiple comparisons (applying a Bonferroni correction) using the functions emmeans, contrast and test of the emmeans package version 1.3.5.1^67^.

## Data Availability

The data supporting the findings of the present study are available from the corresponding author (Yann Coello) upon request.
